# A comparative analysis of teenagers and older pregnant women in the utilization of prevention of mother to child transmission [PMTCT] services in, Western Nigeria

**DOI:** 10.1186/1472-698X-12-13

**Published:** 2012-08-10

**Authors:** Olorunfemi E Amoran, Omotayo F Salami, Francis A Oluwole

**Affiliations:** 1 Department of Community Medicine and Primary Care, College of Health Sciences, Olabisi Onabanjo University Teaching Hospital, Sagamu, Nigeria

## Abstract

**Introduction:**

Most HIV/AIDS infections in women occur at a younger age, during the first few years after sexual debut. This study was therefore designed to assess factors associated with the knowledge and utilization of the prevention of mother-to-child transmission (PMTCT) services by the teenage pregnant women when compared to mature pregnant women in Ogun state, Nigeria.

**Methods:**

This study is an analytical cross-sectional study. A total sample of all pregnant women [52 teenagers and 148 adults] attending the primary health care centres in Sagamu local government area, Ogun State, Nigeria within a 2 months period were recruited into the study.

**Results:**

A total of 225 respondents were recruited into the study. The overall point prevalence of HIV/AIDS infection among those that had been tested and disclosed their result was 4 [2.8%]. The prevalence of HIV among the teenagers was 2 [7.4%] compared with 2 [1.8%] among older women. Only 85 [37.8%] of all respondents were tested through the Voluntary counseling and testing (VCCT) programme and 53 (23.7%) were aware of antiretroviral therapy while 35 (15.6%) have ever used the PMTCT services before.

There was no statistically significant difference in the knowledge of the teenage pregnant women when compared with the older women about mother to child transmission (MTCT) [OR = 1.47, C.I = 0.57-3.95] and its prevention [OR = 0.83, C.I = 0.38-1.84]. The teenagers were 3 times less likely to use the services when compared with the older women. [OR = 0.34, C.I = 0.10-1.00]. Those from the low socio-economic background were about 6 times more likely to utilize PMTCT facilities when compared to those from high socioeconomic background [OR = 6.01, C.I = 1.91-19.19].

**Conclusion:**

The study concludes that the teenage pregnant women who were more vulnerable to HIV/AIDS infection did not utilize PMTCT services as much as the older pregnant women. Special consideration should be given to teenagers and those from high socioeconomic group in the design of scale up programmes to improve the uptake of PMTCT services in Nigeria and other low income countries.

## Background

Sub-Saharan Africa is home to 62% of the worlds’ Human Immunodeficiency Virus (HIV) cases, more than 14 000 people are infected daily with the HIV, and 11 000 people are dying daily due to HIV/AIDS related illnesses [1]. Also, Sub-Saharan Africa is home to 70% of the poorest people in the world. This region has the lowest gross domestic product (GDP) in the world, with more than 60% of the population spending less than US $1 a day [2,3]. An estimated 430,00 new human immunodeficiency virus (HIV) infections occurred among children younger than 15 years of age in 2008, most in sub-Saharan Africa and most due to mother-to-child transmission (MTCT) [4].

In marked contrast, MTCT of HIV has been virtually eliminated in well-resourced settings such as the United States and Europe through the use of combinations of antiretroviral (ARV) drugs for the mother during pregnancy and labor and for the infant postpartum; caesarean delivery to reduce the infant’s exposure to trauma and infection in the birth canal; and formula feeding to protect the infant from transmission from breastfeeding [5]. In the late 1990s, breakthrough clinical trials of shorter and less expensive ARV regimens—a short course of azidothymidine (AZT) for the mother or a single dose of Nevirapine to mother and infant—demonstrated reductions of about 50% in vertical transmission of HIV [5,6]. These advances made prevention of MTCT (PMTCT) feasible in sub-Saharan Africa and other resource-constrained settings. While effective, these interventions are costly and require strong health-care systems.

In Nigeria, HIV prevalence was greater among young women who started having sex at an early age (≤15 years). The HIV prevalence peaked early at 10% among 25–29 year olds [4]. This suggests that most infections in women occur at a younger age, during the first few years after sexual debut. Immature genital tract and cervical ectopy, which is common in young women, might increase the risk. Untreated sexually transmitted diseases may increase the biological susceptibility [4,5]. A vast literature describing randomized, controlled trials clearly demonstrates that interventions with attention to specific elements can be successful in reducing and preventing sexual risk behaviours resulting into HIV/AIDS infection [7-15]. However, teenagers younger than 15 are five times more likely to die during pregnancy or childbirth than women in their twenties and mortality rates for their infants are higher as well. Teenage pregnancy only continues the cycle of poverty [16-18].

This pandemic commonly affects the age group 15 to 29 years. This is largely due to the early age of onset of sexual activity, ignorance of preventive measures and poverty [19-21]. Since over 90% of new HIV infections among infants and young children occur through mother-to-child transmission of HIV, it is obvious that prevention remains the top priority [20]. It is well-documented that focused and well-established interventions for PMTCT have virtually eliminated paediatric HIV in high-income countries, with antenatal care (ANC) playing an important role as a platform for HIV testing and provision of prevention services [22].

PMTCT services received a boost in Nigeria in 2004 when the UNAIDS/WHO recommended routine HIV testing of pregnant women with the right to refuse in order to increase access to PMTCT and ARV therapy in resource-limited countries [23]. Currently, health policies on PMTCT services in Nigeria and Africa has emphasised the importance of preventive care at the PHC level. Coverage of PMTCT service in this region remains low with estimated coverage of 25% on average [24]. This indicates that weak PMTCT services and low coverage rates are leaving mother-to-child transmission of HIV largely unabated and results in high number of new paediatric infections. This study was therefore designed to assess factors associated with the knowledge and utilization of the PMTCT services by the teenage pregnant women when compared to older pregnant women in Ogun state, Nigeria at the PHC level. This has implications in the development of policies that will increase the uptake of PMTCT services and scale up antiretroviral drug uptake among this vulnerable population.

## Methods

### Background of the study area

The study was conducted in Sagamu local government area (SLGA) Ogun state, which is located in the South Western part of Nigeria. Sagamu local government area is one of the 20 local government area in Ogun state. It was carved out of the former Ijebu Remo local government in 1991 and has a total land area of 68.03 km^2^. It is bounded on the west by the Obafemi Owode local government area, on the east by both Ikenne and Odogbolu local government area and also shares a boundary with Ikorodu local government area of Lagos state in the south.

According to the 2006 census, the area has a population of 253,412 inhabitants which consists of mainly remo-speaking people of Ogun state. Other ethnic groups like the Hausas, Igbos and the Benue people are well represented. Most of the towns are either semi-urban or rural. Other major towns in the local government besides Sagamu include Ogijo, Sotubo, Ode-lemo, Emuren and Simawa. The local government has 15 political wards, 12 of which fall within the Sagamu metropolis. This area is a major transit region between the southwest, southeast and the northern part of Nigeria.

There are seven centers for primary health care services and five other health posts spread all over the local government area. There are 52 registered birth attendants and one general and a teaching hospital. As at the time of this study, those primary health care centers that provide antenatal services are located at Ogijo, Sabo and Makun (the other primary health care centers were no longer functional, due to logistic reasons). Conspicuous industrial establishments include the West African Portland Cement (WAPCO), Nulec industries, Sparkwest Nigeria Limited and branches of First bank, Guarantee trust bank, Wema bank and Zenith bank amongst others.

### Study design

This was an analytical cross-sectional study that quantitatively explored the awareness, knowledge and utilization of PMTCT of HIV services by pregnant women. It also compared the knowledge and utilization of PMTCT services among the teenage pregnant women and the older women. All consenting pregnant women in their first pregnancy who attended the PHC centres during the 2 months study period for the first time were recruited into the study in order to assess the utilization of PMTCT services before awareness of pregnancy. The study sought for information prior to ANC attendance.

### Sampling size

The minimum sample size required for the study was estimated to be 138 using the formula

(1) $${{\text{n = Z}}_{\text{α}}}^{2}{\text{pq/d}}^{2},$$

where n is the sample size,

Z_α_ is the standard normal deviate, set at 1.96 (for 95% confidence interval),

d is the desired degree of accuracy (taken as 0.050 and

p is the estimate of our target population having those particular characteristics. MTCT constitute about 10% of the national HIV (i.e. 0.1) burden (FMOH, 2005).

Adjustment for a 10% rate of non-responses and invalid responses yielded a final sample size of 152.

### Data collection

The medical officer of Health/Director of Primary Health care at Sagamu local government secretariat was approached and permission was obtained to conduct the study. Women who consented to take part in the study were interviewed using a structured questionnaire which was administered by trained interviewers. The interviewers were all female medical students rotating through the Community Medicine and primary health care department of the Olabisi Onabanjo University Teaching Hospital during the period of the study and one resident doctor that were involved with the medical care of the study participants. The data were collected on antenatal clinic days by the interviewers at the respective PHC centres. Completed questionnaires were scrutinized on the spot and at the end of the daily field sessions for immediate correction of erroneous entry. Consenting first time pregnant women were interviewed individually over a 10 to 15 minute period in a language they can understand before they were given any health talk. Data were collected over 2 months’ period with the interviewers visiting the centres simultaneously over the study period. (Most of them speak ‘pigeon’ English or Yoruba).

### Study instrument

The instrument was a structured questionnaire consisting of 3 parts, namely:

Section A: includes information on socio-demographic data such as age, marital status, religion, employment status, ethnic group and educational status.

Section B: consists of HIV related knowledge, risk behaviour and safe sexual practices.

Section C: is made up of knowledge and utilization of PMTCT services which includes breastfeeding practices awareness of the means of transmission to the unborn child and where to access help when found to be positive.

Awareness was determined by simply asking such question as:

Can a pregnant woman infected with HIV/AIDS transmit the virus to her unborn child?

Knowledge was determined by such question as:

Mention how transmission of HIV/AIDS from mother-to-child can be prevented? Every respondent that correctly mention one or more ways was classified as knowledgeable.

Utilization of PMTCT services was defined as attendance in any PMTCT service provider center including voluntary counseling and testing [VCCT] prior to presentation for ANC at the PHC centre and was determined by such question such as:

Have you ever presented in any PMTCT center before?

The questionnaire was pretested among 30 women in their first pregnancies receiving antenatal care at primary health care facilities in Ikene local government, a nearby local government to the study area. Appropriate adjustments were then made to the questionnaire to improve its internal validity.

### Criteria for inclusion

Subject must reside within Sagamu local government area (SLGA) of Ogun state.

Subject must be attending the PHC centre for the first time in the present pregnancy.

Subject must not have received prior health talk in the facility before interview

### Ethical consideration

Ethical clearance was obtained from the Olabisi Onabanjo Teaching Hospital Ethics Board. Confidentiality on candidate’s information was maintained. Permission of the State Ministry of Health, HIV/AIDS Control Division was obtained before the commencement of the study.

At each of the selected study site, the matron and medical officer in-charge were informed for consent before the commencement of the study. The purpose, general content and nature of the study were explained to each respondent to obtain verbal and written consent before inclusion into the study.

### Data analysis

The data was entered into SPSS statistical software version 12. Frequencies were generated for detection of errors (data editing). Percentages or means and standard deviation were computed for baseline characteristics of women interviewed. The data analysis focused on univariate frequency table and bivariate cross tabulations that identify important relationships between variables. Respondents were categorized into low and high socioeconomic status using location of resident as cut off. Those from slum areas were categorized as low and those from government reserve areas [GRA] and others were classified as high.

Teenage Pregnancy was as defined by WHO as pregnancy at less than 18 yrs of age.

The relationships between socio-demographic characteristics of the pregnant women and their knowledge and utilization of PMTCT of HIV services were examined through bivariate analysis, by computing odds ratio at 95% confidence level and chi squared and t-tests where appropriate. Predictor variables were restricted to outcome measures that were statistically significant. A p-value ≤ 0.05 or confidence limits which did not embrace unity (1) was considered as statistical significance.

## Results

### Socio-dermographic characteristics of respondents

A total of 225 pregnant women attending the ANC clinics of the primary health care centres for the first time within the study period were recruited into the study. The age of the respondents ranged from 14 to 40 years, (mean 24.34 ± 5.18 years). Majority 175 [77.8%] of the respondents were married with 62.7% being Christians and 37.3% Muslims. Three quarter 170 [75.9%] were of the Yoruba tribe, 10.7% were Hausas and 10.3% were Igbos and 3.1% were from other tribes. About half 111 [49.1%] of the respondents have completed a secondary education, 9.4% had a primary education and 8.5% had no education. Majority 82.7% of the respondents were either in training or employed. Significantly more of the teenagers were pregnant out of wedlock when compared with the older age group [*X*2 = 29.14 p<0.001]. This is shown in Table 1 below.

**Table 1:** Socio-dermographic Characteristics of the Respondents

	**Total**	**Teenage pregnant women**	**Adult pregnant women**	***X*2 [p value]**
**Marital Status**				
Single	50 [22.2]	27 [48.2]	23 [13.6]	29.14 [p<0.001]
Married	175 [77.8]	29 [51.8]	146 [86.4]	
Total	225 [100.0]	56 [100.0]	169 [100.0]	
**Ethnicity**				
Yoruba	170 [80.0]	44 [78.6]	127 [75.1]	3.67 [p = 0.3]
Igbo	24 [10.7]	3 [5.4]	21 [12.4]	
Hausa	23 [10.3]	8 [14.3]	15 [8.9]	
Others	8 [3.1]	1 [1.8]	6 [3.6]	
**Religion**				
Christainity	141 [62.7]	23 [41.1]	118 [69.8]	14.86 [p<0.001]
Islam	84 [37.3]	33 [58.9]	51 [30.2]	
**Level of Education**				
Nil	19 [8.5]	2 [3.6]	17 [10.1]	21.19 [p<0.001]
Primary	21 [9.4]	11 [19.6]	10 [6.0]	
Secondary	110 [49.1]	35 [62.5]	76 [44.6]	
Post -Secondary	74 [33.0]	8 [14.3]	66 [39.3]	
**Occupation**				
Unemployed	39 [17.3]	11 [19.6]	28 [16.6]	37.42 [p<0.001]
Civil Servants	52 [23.1]	1 [1.8]	51 [30.2]	
Traders	55 [24.4]	9 [16.1]	46 [27.2]	
Students	48 [21.3]	20 [35.7]	28 [16.6]	
Apprentices	31 [13.8]	15 [26.8]	16 [9.5]	
**Social Class**				
Low	165 [73.3]	50 [89.3]	115 [68.0]	9.71 [p = 0.008]
High	60 [26.7]	6 [10.7]	54 [31.9]	

#### Knowledge of transmission and prevention of mother to child transmission

About half 33 [58.9%] of the teenage pregnant women and 23 [41.1%] of the mature pregnant women did not know how to correctly use condom to prevent pregnancy and HIV/AIDS infection [OR = 0.57, C.I = 0.29-1.13]. More of the teenage pregnant women 33 [58.9%] know that HIV/AIDS can be transmitted through breastfeeding when compared with the mature pregnant women 94 [55.6%] [OR = 1.14, C.I = 0.59-2.21]. There is no statistically significant difference in the knowledge of the teenage pregnant women when compared with the older women about mother to child transmission (MTCT) [OR = 1.47, C.I = 0.57-3.95] and its prevention [OR = 0.83, C.I = 0.38-1.84]. Furthermore 25.3% of the respondents with more teenagers than older women still believes HIV/AIDS infection should be treated secretly to prevent being discriminated against [OR = 1.77, C.I = 0.87-3.60]. The relationship between knowledge of transmission and prevention of MTCT is shown in Table 2 below.

**Table 2:** Knowledge of transmission and prevention of MCTC by respondents

	**Total**	**Teenage pregnant**	**Older pregnant women**	**Unadjusted odds ratio**
	**No (%)**	**No (%)**	**No [%]**	
	**N = 225**	**N = 56**	**N = 169**	
**Awareness of mother to child transmission of HIV/AIDS**				
Aware	184 [81.8]	48 [85.7]	136 [80.5]	1.47 [0.57-3.95]
Not aware	41 [18.2]	8 [14.3]	33 [19.5]	1.00
**Awareness of HIV Transmission through Breastfeeding**				
Know	127 [56.4]	33 [58.9]	94 [55.6]	1.14 [0.59-2.21]
Don’t know	98 [43.6]	23 [41.1]	75 [44.4]	1.00
**knowledge on correct Condom use for HIV Prevention**				
Yes	153 [68.3]	33 [58.9]	120 [71.4]	0.57 [0.29-2.21]
No	72 [31.7]	23 [41.1]	49 [28.6]	1.00
**Knowledge of Prevention of MTCT**				
Yes	178 [79.1]	43 [76.8]	135 [79.9]	0.83 [0.38-1.84]
No	47 [20.9]	13 [23.2]	34 [20.1]	1.00
**Discrimination against PLWAs [stigma]**				
Yes	57 [25.3]	19 [33.9]	38 [22.5]	1.77 [0.87-3.60]
No	168 [74.7]	37 [66.1]	131 [77.5]	1.00

#### Utilization of PMTCTC services

The overall point prevalence of HIV/AIDS infection among the pregnant women that had been tested and disclosed their result was 2.8%. The prevalence of HIV among the teenagers was 7.4% compared with 1.8% among older women. Among those that had ever been tested, about three quarter [76.0%] of these were tested in the last 6 months and 85 [37.8%] of all the respondents had been tested through the VCCT programme [23.2% of the teenagers and 42.6% of the older women]. However, 26 [16.9%] of those ever tested refused to disclose their test result (8 [22.9%] among the teenagers vs 18 [13.4%] among other respondents). Significantly more of the teenagers were never tested [37.5% vs 20.7%, p<0.001].

Furthermore, 9 (16.1%) of the teenage pregnant women and 44 (26.0%) of mature pregnant women were aware of antiretroviral therapy in the prevention of mother to child transmission [OR = 0.54, C.I = 0.23-1.27]. Only 35 (15.6%) have used the PMTCT services before, with 11.4% of these being teenagers compared with 88.6% of the older women [OR = 0.34, C.I = 0.1-1.00]. Table 3 shows the relationship between utilization of PMTCT services among teenage pregnant women compared with the older women.

**Table 3:** Utilization of PMTCT services by respondents

	**Total**	**Teenage pregnant women**	**Older pregnant women**	**P value/unadjusted odds ratio**
	**No (%)**	**No (%)**	**No (%)**	
	**N = 225**	**N = 56**	**N = 169**	
**Serostatus of Respondents**				
Positive	4 [2.8]	2 [7.4]	2 [1.8]	
Negative	139 [97.2]	25 [92.6]	114 [98.2]	
Refuse to disclose test result [% of total tested]	26 [16.9]	8 [22.9]	18 [13.4]	
Never tested	56 [24.9]	21 [37.5]	35 [20.7]	
**Type of CT done**				
VCCT	85 [37.8]	13 [23.2]	72 [42.6]	
Referred	84 [37.3]	22 [39.3]	62 [36.7]	
Never tested	56 [24.9]	21 [37.5]	35 [20.7]	
**Ever Utilized PMTCT Services**				
Yes	35 [15.6]	4 [7.1]	31 [18.3]	0.34 [0.1-1.00]
No	190 [84.4]	52 [92.9]	138 [81.7]	1.00
**Awareness of ARV Services**				
Yes	53 [23.6]	9 [16.1]	44 [26.0]	0.54 [0.23-1.27]
No	172 [76.4]	47 [83.9]	125 [74.0]	1.00

#### Factors associated with utilization of PMTCT services

Utilization of PMTCT Services was statistically significantly associated with age. The teenagers were 3 times less likely to use the services when compared with the older women. [OR = 0.34, C.I = 0.10-1.00]. Those from the low socio-economic background were about 6 times more likely to utilize PMTCT facilities when compared to those from high socioeconomic background [OR = 6.01, C.I = 1.91-19.19]. There was no statistically significant difference in the use of PMTCT facilities due to Religion [OR = 1.01, C.I = 0.45-2.27], Education [p = 0.47] and Employment status [p = 0.74] as shown in Table 4 below.

**Table 4:** Factors associated with utilization of PMTCT services

	**Total**	**Utilizes PMTCT services**	**Do not utilize PMTCT services**	**Unadjusted odds ratio**
**Marital Status**				
Single	50 [22.2]	5 [14.3]	45 [23.7]	0.54 [0.17-1.57]
Married	175 [77.8]	30 [85.7]	145 [76.3]	1.00
Total	225 [100.0]	35 [15.6]	190 [84.4]	
**Ethnicity**				
Yoruba	170 [80.0]	24 [68.6]	147 [77.2]	1.00
Igbo	24 [10.7]	7 [20.0]	17 [9.0]	2.52 [0.84-7.36]
Hausa	23 [10.3]	3 [8.6]	20 [10.6]	0.92 [0.20-3.63]
Others	8 [3.1]	1 [2.9]	6 [3.2]	1.02 [0.16-6.49]
**Religion**				
Christainity	141 [62.7]	22 [62.9]	119 [62.6]	1.01 [0.45-2.27]
Islam	84 [37.3]	13 [37.1]	71 [37.4]	1.00
**Level of Education**				
Nil	20 [8.9]	4 [11.4]	16 [8.4]	1.25 [0.29-4.99]
Primary	21 [9.3]	1 [2.9]	20 [10.5]	0.23 [0.01-1.93]
Secondary	110 [49.1]	17 [48.6]	93 [49.0]	0.86 [0.36-2.03]
Post -Secondary	74 [32.9]	13 [37.1]	61 [32.1]	1.00
**Occupation**				
Unemployed	39 [17.3]	4 [11.4]	35 [18.4]	1.00
Civil Servants	52 [23.1]	9 [25.7]	43 [22.6]	1.83 [0.46-7.81]
Traders	55 [24.4]	7 [20.0]	48 [25.3]	1.28 [0.30-5.69]
Students	48 [21.3]	9 [25.7]	39 [20.5]	2.02 [0.50-8.65]
Apprentices	31 [13.8]	6 [17.1]	25 [13.2]	2.10 [0.46-10.10]
**Social Class**				
Low	165 [73.3]	26 [74.3]	139 [73.2]	6.01 [1.91-19.19]
High	60 [26.7]	9 [25.7]	51 [26.8]	1.00

## Discussion

The overall point prevalence of HIV/AIDS infection among the pregnant women that had been tested and disclosed their result was 2.8%. The prevalence of HIV among the teenagers was high 7.4% compared with 1.8% among other women. Several studies have also reported similarly higher infection rate among adolescents in Nigeri and sub-saharan Africa [4,25,26], thus suggesting that most infections in women occur at this age, during the first few years after sexual debut. The comparatively low rate of HIV testing among the teenagers reported in this study has also been documented by several studies conducted in sub-Saharan Africa [27,28]. This may remain a major challenge for the PMTCT programme in Nigeria and other developing countries. Innovative approaches to promote their involvement are urgently needed. PMTCT programmes should make clinics and VCCT centres more youth-friendly, and enhance community mobilization and information-education-communication (IEC) activities to promote VCCT among youths.

This study shows that more of the teenagers refuse to disclose their test result and also still discriminate against people living with HIV/AIDS (PLWAs). This may actually be due to fear of being perceived as wayward and the stigma still attached to HIV infection in this semi-rural environment. Routine HIV testing approach is not common in most rural area in sub-Saharan Africa, [27] where HIV infection rates are very high and HIV testing faces considerable barriers, including the fear of stigma and discrimination [29,30]. VCCT should be presented in such a way as to enable the community adopt it as “standard of care” offered to all patients attending a PHC, thereby reducing the risk of stigma and other adverse social consequences attached to its uptake in rural areas [31,32]. Integration of VCCT into other reproductive health services in PHC centers should be encouraged in order to ensure greater coverage.

Awareness of MCTC and its prevention is still comparatively low with no statistically significant difference among the teenagers and the older women in the study population. Although there has been scale-up of PMTCT in many resource-poor settings, ARV treatment programmes have only recently started to become available. Significant advances have occurred in PMTCT [33] in resource-rich settings, perinatal HIV transmission rates are less than 2% due to widespread implementation of prenatal HIV-1 testing, combination antiretroviral treatment during pregnancy, elective caesarean section and avoidance of breastfeeding [34-37]. While effective, these interventions are costly and require strong political will and well organized health-care systems to combat HIV/AIDS epidemics. Scale up programmes should be designed to improve the uptake of PMTCT services especially among this vulnerable group in the low income countries to combat the scourge of HIV/AIDS.

Utilization of PMTCT Services was statistically significantly associated with age. The teenagers were 3 times less likely to use the services when compared with the older women. Those from the low socio-economic background were about 6 times more likely to utilize PMTCT facilities when compared to those from high socioeconomic background. Several studies have reported similar findings [38-43]. This evidence has generated increased interest in the effects of interventions that target the social disadvantage associated with early pregnancy and parenthood. Community sensitization, counseling sessions involving highly motivated community counselors and availability of on-site rapid HIV testing kits may encourage those from high socio-economic background to utilize the PMTCT service centres, this will enhance the prevention MTCT of HIV.

Our study has certain limitations. The study findings are limited in terms of overall generalization and impact since it is not all pregnant women identified in Sagamu local government area that actually deliver in these PHC clinics; most women deliver in other facilities or at home. Despite these limitations, we believe that our data provide useful information for the assessment and implementation of PMTCT services in Nigeria and will also inform policy decision in Nigeria and other low income countries.

## Conclusion

The study concludes that the teenage pregnant women who were more vulnerable to HIV/AIDS infection were less likely to have the knowledge of and utilize PMTCT services when compared to older pregnant women. Special consideration should be given to teenage pregnant women and those from high socioeconomic background in the design of scale up programmes to improve the uptake of PMTCT services especially among this vulnerable group.

## Competing interests

The authors declare that they have no competing interests.

## Authors’ contributions

SOO conceived the study and participated in its design, AOE participated in the analysis and design and also helped to draft the manuscript, FAO participated in the coordination. All authors read and approved the final manuscript.

## Pre-publication history

The pre-publication history for this paper can be accessed here:

http://www.biomedcentral.com/1472-698X/12/13/prepub
